# Sugar coating the invasion: how smut fungi sweet-talk maize cell wall guards

**DOI:** 10.1093/plcell/koag271

**Published:** 2026-09-07

**Authors:** Meenu Singla-Rastogi

**Affiliations:** Assistant Features Editor, The Plant Cell, American Society of Plant Biologists; Department of Biology, Indiana University, Bloomington, IN 47405, United States

Plant pathogens and their hosts are engaged in a perpetual evolutionary arms race. As plants evolve increasingly sophisticated defense mechanisms to prevent infection, pathogens respond with equally ingenious strategies to circumvent them (Peng et al. 2018). The first physical barrier encountered by an invading pathogen is the plant cell wall, a dynamic extracellular structure that provides mechanical support and serves as the primary line of defense. Successful invasion depends on the pathogen's ability to breach the host cell wall (Mohnen 2008; Cosgrove 2024). Although pathogens employ an arsenal of cell wall–degrading enzymes to break down host structural components, this enzymatic breakdown releases damage-associated molecular patterns that trigger plant immune signaling, activate antimicrobial defenses, and induce growth-defense tradeoffs. How biotrophic pathogens remodel the host cell wall for colonization without triggering massive immune activation remains a central question in plant pathology.

Pectin, a major cell wall polysaccharide, is an important determinant of cell wall mechanics and plant immunity. Its susceptibility to enzymatic remodeling depends in part on its methylesterification state. Pectin methylesterases (PMEs), present in plants, bacteria and fungi, catalyze the removal of methyl groups from pectin. Depending on the spatial pattern of demethylesterification, PME activity can either reinforce the wall through calcium-mediated crosslinking or soften it by increasing susceptibility to pectolytic enzymes. To strictly regulate this process, plants produce pectin methylesterase inhibitors (PMEIs) that bind PMEs and suppress their activity. During pathogen attack, this balance dictates the outcome: PMEIs maintain highly methylesterified pectin to fortify the wall barrier, whereas increased PME activity favors cell wall remodeling and pathogen invasion (Pelloux et al. 2007; Wang et al. 2013). Previous work showed that the biotrophic maize smut fungus *Ustilago maydis* increases host PME activity during infection, but how it overcomes PMEI-mediated inhibition was unknown.

In this issue of *The Plant Cell*, Chibbhi Bhaskar and colleagues (Bhaskar et al. 2026) reveal how *U. maydis* uses a secreted effector to manipulate this host pectin regulatory system. *U. maydis* is a biotrophic fungus that relies on living host tissue to form gall-like tumors on aerial maize organs. Using the solopathogenic strain SG200-infected maize seedlings, the authors established that N-glycosylated Effector 1 (Nge1), a conserved 113-amino-acid secreted effector, is required for full virulence. Nge1 was induced during biotrophic growth, and deleting Nge1 reduced disease symptoms. Virulence was fully restored by reintroducing wild-type Nge1, but not by a variant lacking the key N71 N-glycosylation site (Nge1^N71Q^). Subcellular localization confirmed that Nge1 localizes to the apoplast, placing it in the extracellular compartment where pectin remodeling occurs. Together, these results established both the biological importance and the site of action of Nge1, providing a foundation for subsequent investigation of its molecular targets.

To determine how Nge1 acts in the apoplast, the authors sought host interacting partners. Using glycosylated Nge1 and the nonglycosylated Nge1^N71Q^ variant as baits to capture proteins from apoplastic fluid isolated from *U. maydis*-infected maize leaves, followed by liquid chromatography–tandem mass spectrometry and protein-interaction assays, they identified PMEI45 and the inhibitory PRO domain of PME19 as primary targets. Subsequent screens revealed that Nge1 also recognizes PMEI46 and the PRO domain of PME20. Importantly, N-glycosylation distinctly modulated these interactions: glycosylated Nge1 interacted efficiently with PMEI45, whereas nonglycosylated variants showed markedly reduced affinity. In contrast, interactions with PMEI46 and PRO19 were largely glycosylation independent. Thus, the N-glycosylation specifically expands Nge1's target spectrum to include the abundant host inhibitor PMEI45.

The authors next investigated the biological consequences of these interactions. Transient co-expression in *Nicotiana benthamiana* demonstrated that PMEI45 and PMEI46 naturally inhibit host PME activity by binding the catalytic domains of maize PME19 and PME20. Glycosylated Nge1 effectively weakened the PMEI45–PME19 interaction, relieving PME suppression far more efficiently than the nonglycosylated counterpart (Fig. 1). In maize, leaf cross-sections expressing Nge1 contained significantly higher levels of demethylesterified pectin and reduced levels of methylesterified pectin compared to controls. Consistently, total PME activity was higher in maize leaves infected with wild-type or Nge1-complemented *U. maydis* than in those infected with the Δnge1 or Nge1^N71Q^ strains. Enhanced PME activity further accelerated host pectolytic activity, particularly polygalacturonase activity, and overexpression of either Nge1 or PME19 increased host susceptibility. These findings support a model where Nge1 neutralizes host PMEIs to unleash host PMEs, driving host-mediated pectin demethylesterification and loosening without relying on fungal secreted degrading enzymes.

**Figure 1:**
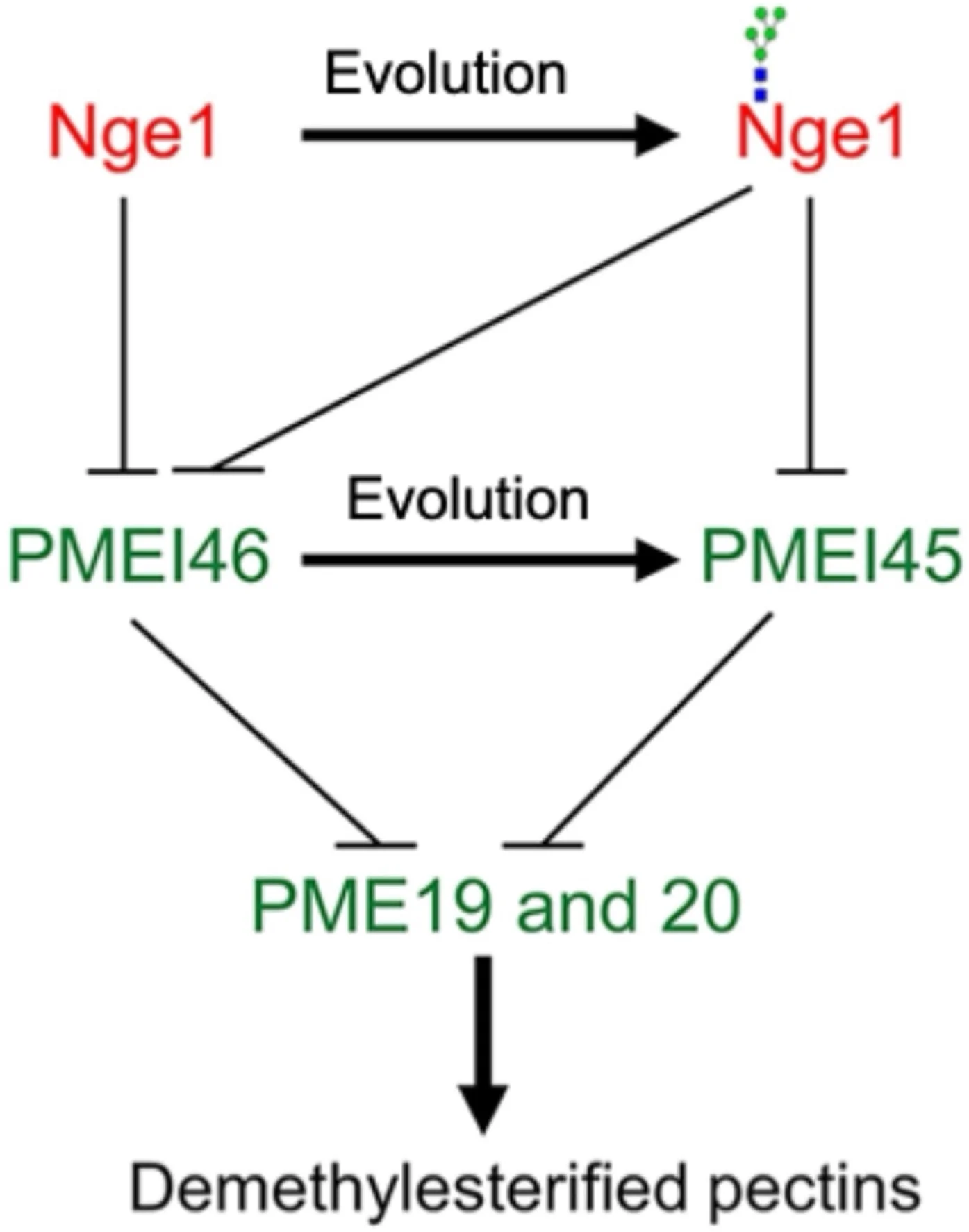
Model for Nge1-mediated cell-wall remodeling during *Ustilago maydis* infection. N-glycosylated Nge1 interacts with maize PME146 and PMEI45, relieving inhibition of PME19 and PME20 and promoting pectin demethylesterification and cell-wall loosening. Adapted from Fig. 7 of Bhaskar et al. (2026).

This mechanism also provides a compelling evolutionary explanation for glycosylation-dependent effector targeting. PMEIs are thought to have evolved through duplication and divergence of the inhibitory PRO domains of type-I PMEs (Wang et al. 2013). Bhaskar et al proposed that host PMEI diversification generated inhibitors such as PMEI45 that evade recognition by ancestral nonglycosylated Nge1 effectors, creating a new barrier to fungal colonization. In response, N-glycosylation of Nge1 emerged as a counteradaptation, restoring binding to PMEI45 while preserving affinity for other PMEIs (Fig. 1). Supporting this evolutionary model, a naturally nonglycosylated Nge1 ortholog from *Ustilago hordei* failed to rescue the *U. maydis* Δnge1 virulence defect in maize, whereas engineering an N-glycosylation site into the *U. hordei* ortholog restored both glycosylation and virulence. Structural modeling further suggests that the glycan alters the local surface charge properties and constrains a flexible C-terminal region of Nge1, potentially preconfiguring the effector for optimal PMEI45 engagement.

Bhaskar and colleagues connect 3 distinct tiers of host-pathogen co-adaptation: N-glycosylation of an apoplastic effector, selective neutralization of host PME inhibitors, and host-driven pectin remodeling. Rather than merely enhancing protein stability, N-glycosylation acts as a molecular switch that fine-tunes effector specificity and enables *U. maydis* to exploit the host PMEI-PME regulatory network. This “*inhibitor-of-an-inhibitor*” strategy offers a biochemical route for a biotroph to loosen the host cell wall while avoiding the massive secretion of fungal cell wall–degrading enzymes that would otherwise trigger host immunity. More broadly, the study highlights post-translational glycosylation as an evolutionary mechanism for expanding effector function and identifies pectin methylesterification as a potential leverage point for engineering disease-resistant maize.

## Recent related articles in *The Plant Cell*

Xie et al. 2024 describes a transcriptional regulatory module comprising transcription factors ZINC FINGER FAMILY PROTEIN5 (ZAT5), and BEL1-LIKE HOMEODOMAIN2 (BLH2) and BLH4, that functions in modulating the demethylesterification of homogalacturonan in seed coat mucilage.

Rastogi et al. 2025 demonstrate that the plasma membrane-localized GDSL esterase/lipase AtGELP53 in *Arabidopsis thaliana* controls xyloglucan and xylan deacetylation, driving cell wall remodeling and activating pathogen resistance through oligosaccharide-mediated signaling.

## Data Availability

No new data were generated or analyzed in support of this research.
